# Production of iron-enriched yeast and it’s application in the treatment of iron-deficiency anemia

**DOI:** 10.1007/s10534-024-00592-3

**Published:** 2024-03-28

**Authors:** Ying Chen, Yuanxiang Pang, Hongbing Wan, Xinyi Zhou, Mingli Wan, Shengshuo Li, Xuelian Liu

**Affiliations:** State Key Laboratory of Forage Microbiology Engineering, Beijing Da Bei Nong Group, Beijing, 100194 China

**Keywords:** Iron deficiency anemia, Mice, *S. cerevisiae* enriched iron, Hematological parameters, Antioxidant activity

## Abstract

Iron deficiency anemia (IDA) is one of the most serious forms of malnutrition. Wild type strains of *Saccharomyces cerevisiae* have higher tolerance to inorganic iron and higher iron conversion and accumulation capacity. The aim of this study was to investigate the effect of *S. cerevisiae* enriched iron as a potential organic iron supplement on mice with iron deficiency anemia. 60 male Kunming mice (KM mice, with strong adaptability and high reproduction rate, it can be widely used in pharmacology, toxicology, microbiology and other research) were randomly divided into normal control group and iron deficiency diet model group to establish IDA model. After the model was established, IDA mice were randomly divided into 5 groups: normal control group, IDA group, organic iron group (ferrous glycinate), inorganic iron group (ferrous sulfate) and *S. cerevisiae* enriched iron group. Mice in the experimental group were given different kinds of iron by intragastric administration once a day for 4w. The results showed that *S. cerevisiae* enriched iron had an effective recovery function, and the body weight and hematological parameters of IDA mice returned to normal levels. The activities of superoxide dismutase, glutathione peroxidase and total antioxidant capacity in serum were increased. In addition, the strain no. F8, able to grow in an iron-rich environment, was more effective in alleviating IDA and improving organ indices with fewer side effects compared to ferrous glycinate and ferrous sulfate groups. This study suggests that the iron-rich strain no. F8 may play an important role in improving IDA mice and may be developed as a new iron supplement.

## Introduction

Iron is an essential trace element for most life forms and is involved in many biological processes (Dallman 2003). Iron deficiency is one of the most common nutritional deficiencies worldwide. Severe iron deficiency can cause iron deficiency anemia (IDA), and iron deficiency can impair immune response (Hassan et al. 2016), physical work ability and intellectual function (Duque et al. 2014).

Iron ion is the second most abundant metal element in the Earth’s crust, but iron deficiency, especially iron deficiency anemia, is a common nutrient deficiency in humans, affecting about 1.6 billion people worldwide (Benoist et al. 2008). Iron plays a crucial role in the normal development of the central nervous system, which can affect myelin formation, neuronal and glial energy metabolism, and neurotransmitter production (Lozoff et al. 2006). Iron deficiency during the critical period of development will have irreversible harmful effects on the brain. Lead to cognitive and behavioral abnormalities (Georgieff 2011).

To improve iron deficiency anemia, several forms of iron supplements have been developed over the past two decades. At present, the research on iron supplements is more concerned about the blood-replenishing activity of iron supplements, and the side effects of iron supplements such as intestinal inflammation are still lacking.

At present, oral iron supplements used in clinical practice are mostly traditional iron supplements, such as ferrous sulfate (FeSO_4_), ferrous succinate, ferrous fumarate, ferrous gluconate, ferrous glycine (Fe-Gly), etc., but they all have iron odor, instability, low bioavailability, and are prone to dietary taboo, free iron toxicity, gastrointestinal discomfort and other symptoms (Wang et al. 2016).

FeSO_4_ was first used in clinical treatment, easily soluble in water, good bioavailability, low cost, is the most widely used oral iron agent. However, its bioavailability is low, accompanied by side effects such as stomach pain and diarrhea, and it is easy to cause lipid peroxidation and even DNA damage (Dewey et al. 2002; Hyder et al. 2002) FeSO_4_ has high iron content and good absorption effect, which may help alleviate the symptoms of iron deficiency and anemia to a certain extent. But it can cause many adverse reactions, such as diarrhea and constipation (Souza et al. 2009). Other common inorganic iron agents include FeCl_3_·6H_2_O, FeCO_3_, FeNaPO_4_ and Fe_2_P_2_O_7_.

Organic ferric agents are mainly some small molecular organic acid ferric salts, such as ferrous fumarate (43.4%), ferric albuminate (7%) and Fe-Gly (Cancelo-Hidalgo et al. 2013). Studies have shown that Fe-Gly iron is better absorbed than FeSO_4_ iron (Bovellbenjamin et al. 2000). However, recently, many new iron supplements have appeared, such as the chelation of iron with peptides derived from proteins in hydrolyzed foods, and the conversion of iron into organic iron by cells (Chaud et al. 2002; Miquel and Farré 2007). There are also some new macromolecular complexes such as polypeptide iron chelates, heme iron, polysaccharide iron, iron-rich yeast, etc. These have caused great concern.

Among them, iron-rich yeast has the characteristics of high absorption rate, low irritation and rich nutrition. Inorganic iron can be converted into organic form through the yeast reproduction process, which makes iron more easily absorbed by the body. It is a good new iron supplement for preventing and treating IDA. Studies have shown that iron-rich bread yeast can improve the levels of red blood cells (RBC) and hemoglobin (HGB) in anemic mice, and can alleviate the damage caused by anemia to various organs of the body (Kyyaly et al. 2015). The form of iron in iron-rich yeast is similar to that in human body, so iron-rich yeast does not have iron toxicity caused by free iron ion enrichment. Studies have shown that compared with FeSO_4_ and amino acid chelated iron, iron-rich yeast has a higher bioavailability, showing a good effect on IDA treatment (Geng 2014).

In conclusion, the treatment of IDA should increase iron intake and iron absorption and availability, reduce intestinal adverse reactions as much as possible, and thus improve the effectiveness of treatment and patient compliance. Therefore, the aim of this study was to investigate the improvement of the *S. cerevisiae* strain no. F8 enriched iron (Fe-F8) on iron deficiency anemia mice. The effects of Fe-F8 on hematological parameters and organs of mice were studied. In addition, the antioxidant effects of Fe-F8 on anemic mice were evaluated to investigate whether Fe-F8 is promising for development as a new iron supplement.

## Materials and methods

### Preparation of iron-enriched *S. cerevisiae*

#### Test tube culture seed solution

Iron-enriched *S. cerevisiae* was immersed in a test tube containing 10 mL YPD medium (2% glucose, 1% yeast powder, 2% tryptone) at 30 °C and 200 rpm for 18 h. Then, it was inoculated in 250 mL flask of 50 mL YPD medium at a ratio of 2% (v/v) at 30 °C, 200 rpm, and oscillated for 18 h as seed liquid for reserve.

#### Flask culture

The iron-enrich *S. cerevisiae* seed liquid was inoculated in 250 mL flask of 50 mL YPD medium at a ratio of 2% (v/v) at 30 °C and 200 rpm, and oscillated for 18 h as secondary seed liquid.

#### Fermentation culture

##### Molasses medium

Molasses 2%, (NH_4_)_2_SO_4_ 0.6%, KH_2_PO_4_ 1.0%, FeSO_4_·7H_2_O 5 mM, pH 5.1.

##### CDM medium

Pancreatic digest of casein 1.7%, Papaic digest of soybean meal 0.3%, Sodium choride 0.5%, Dipotassium hydrogen phosphate 0.25%, C_6_H_12_O_6_·H_2_O 0.25%, pH 7.3.

The secondary seed solution was added to the fermentation medium at 10% (v/v). Add iron salt before inoculation, take 36 mL of 1.5 L fermentation solution and add it into culture solution. The fermentation time was 28 h. Fermentation temperature 30 °C, rotational speed: 0–2 h: 200 rpm; 2–5 h: 300 rpm; 5–8 h: 400 rpm; 8–10 h: 500 rpm; 10–12 h: 600 rpm; After 12 h: 700 rpm; If the dissolved oxygen is less than 30%, the speed is adjusted to 800 rpm. Maintain fermentation pH at 4.4 with 20% NaOH solution. The dissolved oxygen is controlled at 50–60%.

### Cell iron content evaluation

After fermentation was completed, the iron-enriched *S. cerevisiae* cells with a dry weight of about 0.1 g were weighed into a digestion bottle, and 5 mL of digestion liquid (HNO_3_:HClO_4_ = 5:1) was added to the heating plate at 200 °C for digestion until the liquid was clear and transparent. Then, the cells were removed and cooled, and the volume of distilled water was fixed to 25 mL, and 1 mL of constant volume liquid was absorbed and 4 mL of distilled water was added. After mixing well, absorb 200 μL into 7 mL EP tube, add 3.0 mL distilled water, add 0.1 mL 1 M hydrochloric acid solution, 0.1 mL 10% hydroxylamine hydrochloride, 0.1 mL 0.12% o-diazophene (1, 10-phenanthroline), add 0.5 mL 10% sodium acetate, shake well. The content of cell iron was calculated at 510 nm by using the absorbance value of the blank solution without ferriferous agent as reference solution.

### Animals and experimental design

#### Animals

The 3-week-old healthy male KM mice were from Beijing Weitonglihua Laboratory Animal Technology Co., LTD. According to the requirements of the National Act on the use of laboratory animals (People’s Republic of China), all animals are used in the current research after the evaluation and accreditation of the Association for the Care of Laboratory Animals (AAALAC) and the protocol for Animal testing is approved by the Animal Ethics Committee. All mice were housed in stainless steel cages with sawdust pads and kept in an environmentally controlled room (24 ± 2 °C and 50 ± 10% relative humidity) for a 12-h light/dark cycle. The sawdust mat is renewed every 3 days and the rats have free access to food and water.

#### Experimental design

After 3 days of adaptation, 60 3-week-old male KM mice were randomly divided into normal control group (n = 12) and model group (n = 48). The normal control group was fed the standard diet of AIN 93G standard feed (Ingredients: casein, corn starch, corn dextrin, sugar, cellulose, soybean oil, methionine, choline, vitamins, minerals, TROPHIC Animal Feed High-tech Co., Ltd, Nantong, China) throughout the experiment. The model group was AIN-93G iron deficient feed (containing 3–8 mg iron per kg diet, TROPHIC Animal Feed High-tech Co., Ltd,Nantong, China) induced IDA model. The whole experiment process was strictly controlled, iron staining was avoided, and hemoglobin (Hb) levels were analyzed weekly. After 4 weeks, IDA with Hb content less than 90 g/L was taken as IDA, and then recovery experiment was carried out.

After IDA mouse model was established, IDA mice (n = 48) were randomly divided into 4 groups with 12 mice in each group. Hb concentration was balanced. IDA iron deficiency model group was given the above iron deficiency diet. Organic iron group was given 3.0 mg Fe/kg BW Fe-Gly; the inorganic iron group was given 3.0 mg Fe/kg BW FeSO_4_, and the sample group was given 3.0 mg Fe/kg BW *S. cerevisiae* enriched iron. Fe-Gly, FeSO_4_ and Fe-F8 were dissolved in distilled water, and the mice were given the above dosage by intragastric administration. The normal control group and IDA model group were given the same volume of deionized water. All supplements were prepared fresh before use and administered by gavage at 10 am daily for 4 weeks. During this time, weigh yourself once a week.

#### Sample collection

At the end of the whole experiment, the weight of the mice was measured after fasting for 12 h, the mice were dissected, and blood was collected and placed in the heparin sodium anticoagulant tube. The collected samples were centrifuged to obtain plasma and serum and frozen at − 20 °C for further analysis. All mice, heart, liver, stomach, kidney, spleen, and colon, were rinsed with normal saline, weighed, and stored at − 80 °C for further analysis.

#### Hematological test

The hemoglobin content (Hb), red blood cell count (RBC) and hematocrit (HCT) in the blood were measured by the kit. HCT is expressed as the percentage of red blood cells in total blood volume. Serum iron (SI) concentration was measured using the SI test kit (Beijing Solebol Technology Co., LTD., Beijing, China) according to the manufacturer’s instructions.

#### Organ coefficient

Pathological examination of mice was performed by naked eye during dissection. All mice had their hearts, livers, stomachs, kidneys, spleens and colons removed and weighed. The relative weight of each organ is calculated based on the final weight measured that day. The organ coefficient is calculated as follows:

$${\text{Organ coefficient}} = {\text{organ weight}}/{\text{rat body weight}} \times {1}00$$

#### Histopathology study

The colon and liver of the same size were cut out at the same location and placed in 4% paraformaldehyde fixing solution, which was used to make tissue sections. After paraffin embedding and hematoxylin–eosin staining, the tissue morphology of the colon and liver was observed by optical microscope magnified by 200 times.

### Determination of antioxidant enzyme activity and malondialdehyde level and total antioxidant capacity

Serum glutathione peroxidase (GSH-PX) and superoxide dismutase (SOD) activity and malondialdehyde (MDA) content and total antioxidant capacity (T-AOC) were measured using a diagnostic kit (Beijing Solaibo Technology Co., LTD., Beijing, China) according to the manufacturer’s instructions.

### Statistical analysis

Data are expressed as mean ± SD and analyzed by one-way analysis of variance (ANOVA) and Duncan’s multiple range tests using SPSS 17.0 software. p < 0.05 was considered statistically significant.

## Results and discussion

### Preparation and determination of iron content in *S. cerevisiae*

Strain no. F8 was selected according to its biomass and enrichment rate of iron ions. Studies have shown that iron ion can inhibit the growth of microorganisms (Zhang et al. 2015), so in order to obtain strains with high iron and high biomass, it is necessary to select the best concentration of iron ion in the medium.

As shown in Table 1, Strain no. F8 has its unique growth characteristics, with high biomass and cell iron content in molasses medium. Although the iron-rich strains in CDM medium had higher cell iron content, their biomass was low, and CDM medium was not suitable for large-scale fermentation due to its complex composition, cumbersome configuration and high cost, so molasses medium was used for further optimization. Furthermore, the effects of adding FeSO_4_·7H_2_O or triferric citrate in molasses medium with different concentration gradients on cell growth and iron enrichment of iron-enriched strain no. F8 were investigated.

**Table 1:** Screening results of *S. cerevisiae* enriched iron

Strain	CDM medium		Molasses culture medium	
	Biomass (g/L)	Cell iron content (mg/g)	Biomass (g/L)	Cell iron content (mg/g)
F1	8.6 ± 0.1	1.05 ± 0.03	9.1 ± 0.1	0.99 ± 0.05
F2	9.0 ± 0.1	1.21 ± 0.04	10.1 ± 0.1	1.06 ± 0.04
F3	8.4 ± 0.1	1.46 ± 0.03	9.7 ± 0.4	1.31 ± 0.05
F4	8.7 ± 0.1	1.10 ± 0.03	9.9 ± 0.3	1.03 ± 0.05
F5	7.8 ± 0.1	1.35 ± 0.03	9.6 ± 0.1	1.30 ± 0.06
F6	9.6 ± 0.2	1.15 ± 0.05	11.8 ± 0.3	0.99 ± 0.03
F7	6.8 ± 0.1	1.32 ± 0.04	8.5 ± 0.1	1.27 ± 0.05
F8	11.2 ± 0.3	1.62 ± 0.06	13.0 ± 0.3	1.49 ± 0.06
F9	7.1 ± 0.1	1.16 ± 0.05	8.9 ± 0.1	1.09 ± 0.04

*Strain no. F1-F9 was obtained from lab-preserved strains. Molasses medium: molasses 2%, (NH_4_)_2_SO_4_ 0.6%, KH_2_PO_4_ 1.0%, FeSO_4_·7H_2_O 5 mM, pH 5.1; CDM medium: Pancreatic digest of casein 1.7%, Papaic digest of soybean meal 0.3%, Sodium choride 0.5%, Dipotassium hydrogen phosphate 0.25%, C_6_H_12_O_6_·H_2_O 0.25%, pH 7.3

The experimental results in Table 2 show that the biomass of iron-rich strains supplemented with FeSO_4_·7H_2_O and triferric citrate has little difference, but the iron content of cells supplemented with FeSO_4_·7H_2_O is higher than that of cells supplemented with triferric citrate. Strain no. F8 can grow in a high iron environment. Finally, the highest biomass and cell iron content were obtained in the medium with 12 mM FeSO_4_·7H_2_O concentration.

**Table 2:** Iron enrichment results of iron-enriched strain no. F8 in molasses medium with different iron concentrations

Iron concentration (mM)	Biomass (g/L)		Cell iron content (mg/g)	
	FeSO_4_	Ferric citrate	FeSO_4_	Ferric citrate
2	9.53 ± 0.46	10.39 ± 0.19	1.83 ± 1.75	0.78 ± 0.29
4	10.56 ± 0.17	10.99 ± 0.14	6.69 ± 5.61	2.80 ± 0.08
6	11.87 ± 0.24	10.64 ± 0.14	12.75 ± 3.66	6.82 ± 0.07
8	11.69 ± 0.37	10.84 ± 0.02	16.64 ± 0.61	9.50 ± 0.16
10	11.90 ± 0.14	11.30 ± 0.24	19.96 ± 4.05	12.37 ± 0.65
12	12.31 ± 0.53	12.30 ± 0.39	21.10 ± 7.20	16.35 ± 0.46

In this study, the genome of iron-rich Strain no. F8 was extracted, ITS sequence was amplified, and the strain was identified as *Saccharomyces cerevisiae* by re-comparison in the database.

Yeast is a good carrier of trace elements, which can enrich many trace elements including iron. By means of fermentation technology, adding an appropriate amount of iron ion to the medium can make yeast cells absorb a large amount of iron ion during the proliferation process and bind it to the organic parts of the cells, thus obtaining iron-rich yeast (Zhang et al. 2004). Relevant studies on iron-rich yeasts have been carried out both at home and abroad, and most of them used *Saccharomyces cerevisiae* as the carrier of iron enrichment (Yuan et al. 2004), In addition, other researchers have used *Rhodotorula glutinis* (Xue et al. 2003), *Candida intermedia*, *Kluyveromyces marxianus* (Paš et al. 2007) and so on. Iron-rich yeast can convert inorganic sources of iron into low-toxicity, bioavailable organic iron, which is more beneficial to the human body and has higher biological activity, and is suitable as a new safe iron supplement for the prevention and treatment of iron deficiency anemia (Yuan et al. 2004). In addition, yeast is rich in amino acids, proteins, vitamins and other nutrients (Paš et al. 2007), and can also be used as a nutritional element supplement, energy and immune enhancer in addition to iron supplementation (Zhang et al. 2021).

### Body weight change in mice

**Fig. 1:**
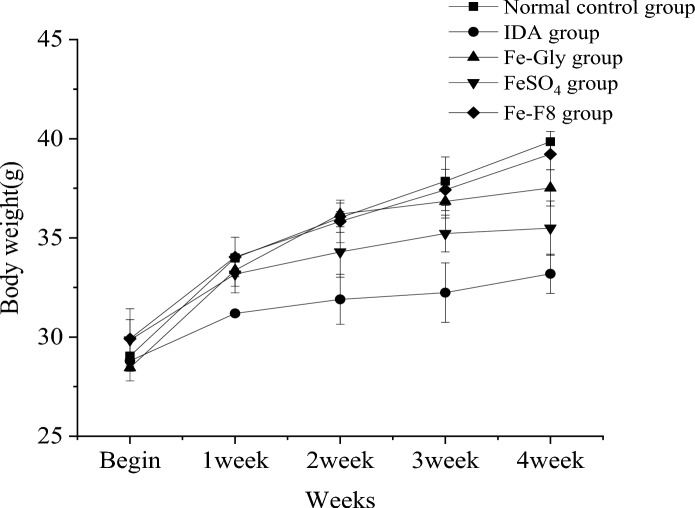
Changes of body weight in different groups of mice. The IDA group was not treated, normal control group was treated with standard granule diet, Fe-Gly group was treated with ferrous glycine (3 mg iron), FeSO_4_ group was treated with ferrous sulfate (3 mg iron), and Fe-F8 group was treated with yeast iron (3 mg iron), respectively

Figure 1 shows the growth of mice in each group. There was a large difference in weight gain. The feed intake of the model group was high but the weight of the model group increased more slowly than that of the normal control group, indicating that iron deficiency affected the normal growth of mice. Zheng’s study also obtained similar results (Zheng et al. 2001). The Fe-F8 group was able to gain weight significantly, which was comparable to the normal control group. However, the overall weight gain trend decreased, which may be due to the small stress response of mice in the early stage, and the weight of mice in the late stage also tended to be saturated. Among them, Fe-Gly group and FeSO_4_ group lost weight in the later period, and the ratio of feed to gain was significantly higher than other groups. Table 3 survival rate showed that the FeSO_4_ group died the most, 5 mice in total, and more lesions were found in their organs after dissection. Other studies have also shown that FeSO_4_ has certain toxicity, which may be detrimental to mouse growth (Souza et al. 2009). Many studies on IDA have shown that IDA affects growth in rats, so our results show that these findings are consistent with previous reports (Fu-Rong et al. 2014).

**Table 3:** Effects of different treatments on survival rate of mice

	Normal control group	IDA group	Fe-Gly group	FeSO_4_ group	Fe-F8 group
Death	0	0	1	5	0
Survival rate (%)	100%	100%	92%	58%	100%
Time of death	–	–	Day 14 after Fe-Gly	After FeSO_4_ administration, 1 died on day 4, 2 died on day 7, and 2 died on day 14	–

*The IDA group was not treated, normal control group was treated with standard granule diet, Fe-Gly group was treated with ferrous glycine (3 mg iron), FeSO_4_ group was treated with ferrous sulfate (3 mg iron), and Fe-F8 group was treated with yeast iron (3 mg iron), respectively

### Changes of organ coefficient in mice

As shown in Table 4, the heart coefficient in IDA group was significantly higher than that in Normal control group (p < 0.05). In addition, after supplementation of Fe-Gly, FeSO_4_ and Fe-F8, the heart coefficient showed some improvement, this result was also observed by Zhang et al. (2016). In iron-deficient organisms, liver mass and volume are significantly reduced, which is typical of iron deficiency. The organ coefficients of the livers in iron enriched supplemented animals were lower than that of iron-deficient animals. Table 4 Organ index showed that spleen weight of mice in iron deficiency group was significantly higher than that in other groups (p < 0.05). This result was also observed by Yun et al. (2011).Kidney index showed no significant difference, and some studies showed that iron deficiency had no significant effect on spleen weight.

**Table 4:** Organ coefficient in the heart, liver, stomach, kidney and spleen of mice in different groups

Group	Organ coefficient				
	Heart	Liver	Stomach	Kidney	Spleen
Normal control group	0.53 ± 0.06^d^	5.31 ± 0.41^a^	1.57 ± 0.48^c^	1.66 ± 0.29^a^	0.29 ± 0.07^ab^
IDA group	0.59 ± 0.08^a^	5.03 ± 0.42^a^	1.83 ± 0.13^b^	1.64 ± 0.18^a^	0.31 ± 0.08^a^
Fe-Gly group	0.56 ± 0.01^c^	5.01 ± 0.39^c^	2.15 ± 0.06^a^	1.51 ± 0.19^a^	0.26 ± 0.06^c^
FeSO_4_ group	0.55 ± 0.08^c^	4.79 ± 0.40^d^	2.13 ± 0.36^a^	1.51 ± 0.09^a^	0.19 ± 0.05^d^
Fe-F8 group	0.57 ± 0.11^b^	5.19 ± 0.59^b^	1.70 ± 0.69^bc^	1.58 ± 0.21^a^	0.28 ± 0.06^bc^

*The superscript of the data in the table was obtained through ANOVA test analysis, and different letter superscripts indicated significant differences in the measured substance content (p < 0.05). The IDA group was not treated, normal control group was treated with standard granule diet, Fe-Gly group was treated with ferrous glycine (3 mg iron), FeSO_4_ group was treated with ferrous sulfate (3 mg iron), and Fe-F8 group was treated with yeast iron (3 mg iron), respectively

### HE section of colon and liver tissue

**Fig. 2:**
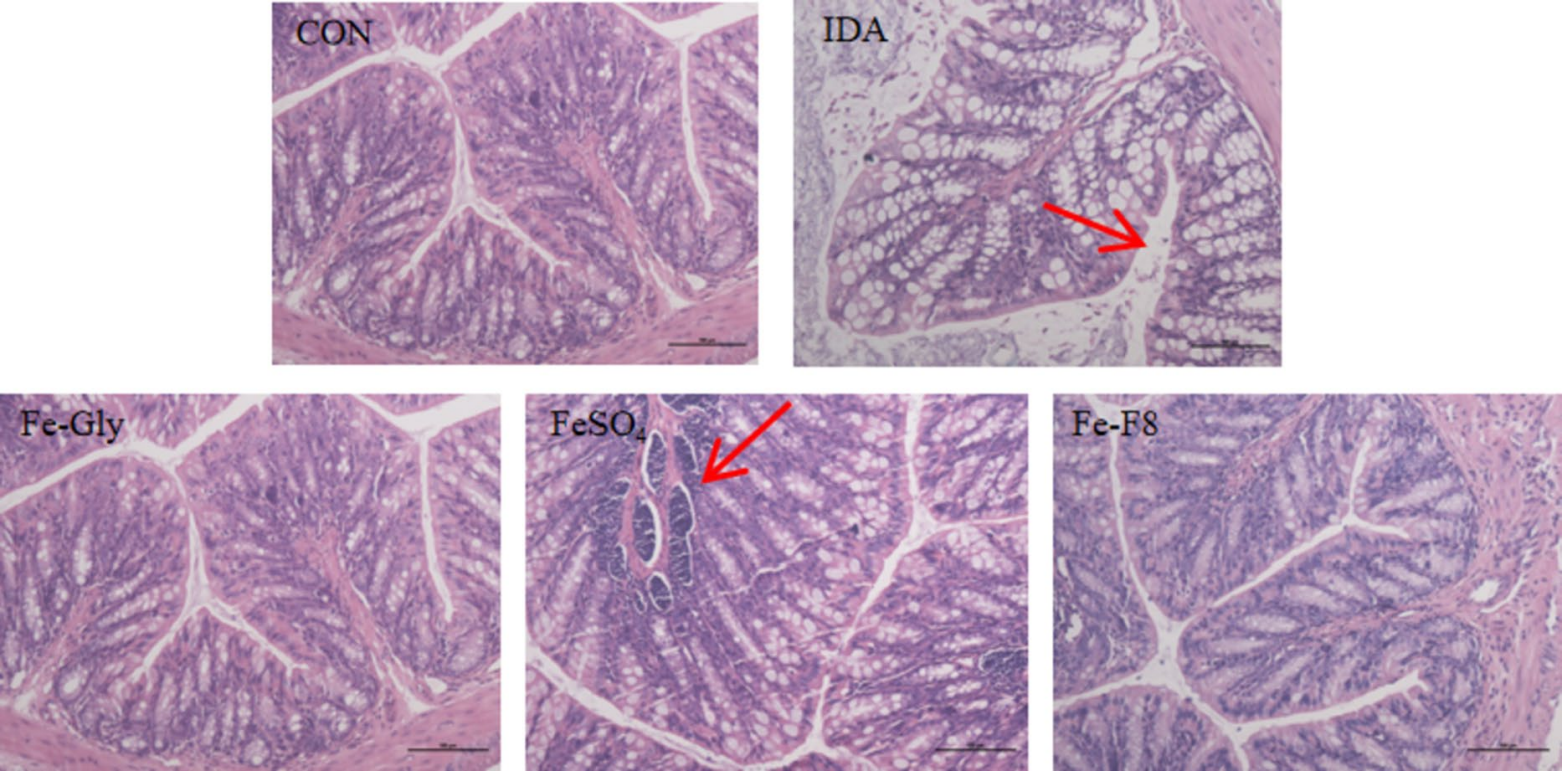
Colon tissue morphology of mice in each group (H&E staining, 200× magnification)

The intestinal barrier plays an important role in the health of the body as it is the main immune, digestive and nutrient absorbing tissue. Colon tissue sections were obtained by H&E staining to further study the effect of iron supplementation on the intestinal tract of IDA mice. As shown in Fig. 2, the mucosal epithelial cells of the colon tissue of mice in the normal control group were closely arranged and orderly, the lamina propria was intact, and the submucosal tissue was intact, with a large number of well-structured, long and clear crypts and villi, and the muscle fibers were neatly arranged and no lesions occurred. Compared with the normal control group, the colonic tissue of mice in IDA group had obvious changes in the overall structure, including mucosal epithelial cell arrangement defect, lamina propria atrophy, crypt structure damage, and epithelial barrier deformation. Wang et al. showed similar results in the colons of IDA mice (Wang et al. 2019), IDA leading to structural abnormalities and dysfunction of the organism biofilm, causing damage and inflammation of colon epithelial cells.

After 4w of intragastric administration, part of the acines in the colon tissue of the FeSO_4_ group showed necrosis and defect and lamina propria atrophy, but the submucosal tissue was intact, the muscle fibers were arranged neatly and no lesions occurred. The colonic tissue structure of mice in Fe-Gly group and Fe-F8 group was orderly, the mucosa tended to be normal, the lamina propria was intact, the crypt structure basically returned to normal, inflammatory cell infiltration basically disappeared, the gap between the mucosa and the lower layer was significantly reduced, and the muscle fibers were neatly arranged without any lesions. These results indicate that supplementation of a certain dose of iron can effectively improve the intestinal mucosal barrier damage and inflammatory cell infiltration caused by IDA, among which Fe-Gly and Fe-F8 have better improvement effects than FeSO_4_ at the same dose. Wang’s studies on other peptide iron chelates have also obtained similar results (Wang 2013), In the FeSO_4_ group, intestinal tissue was damaged, a large number of mucosal epithelial cells were shed, villi erosion was observed, intestinal mucosa in the Marine fish skin peptide iron group, casein peptide iron group and whey protein group was improved, and the villi were intact and the glands were normal.

**Fig. 3:**
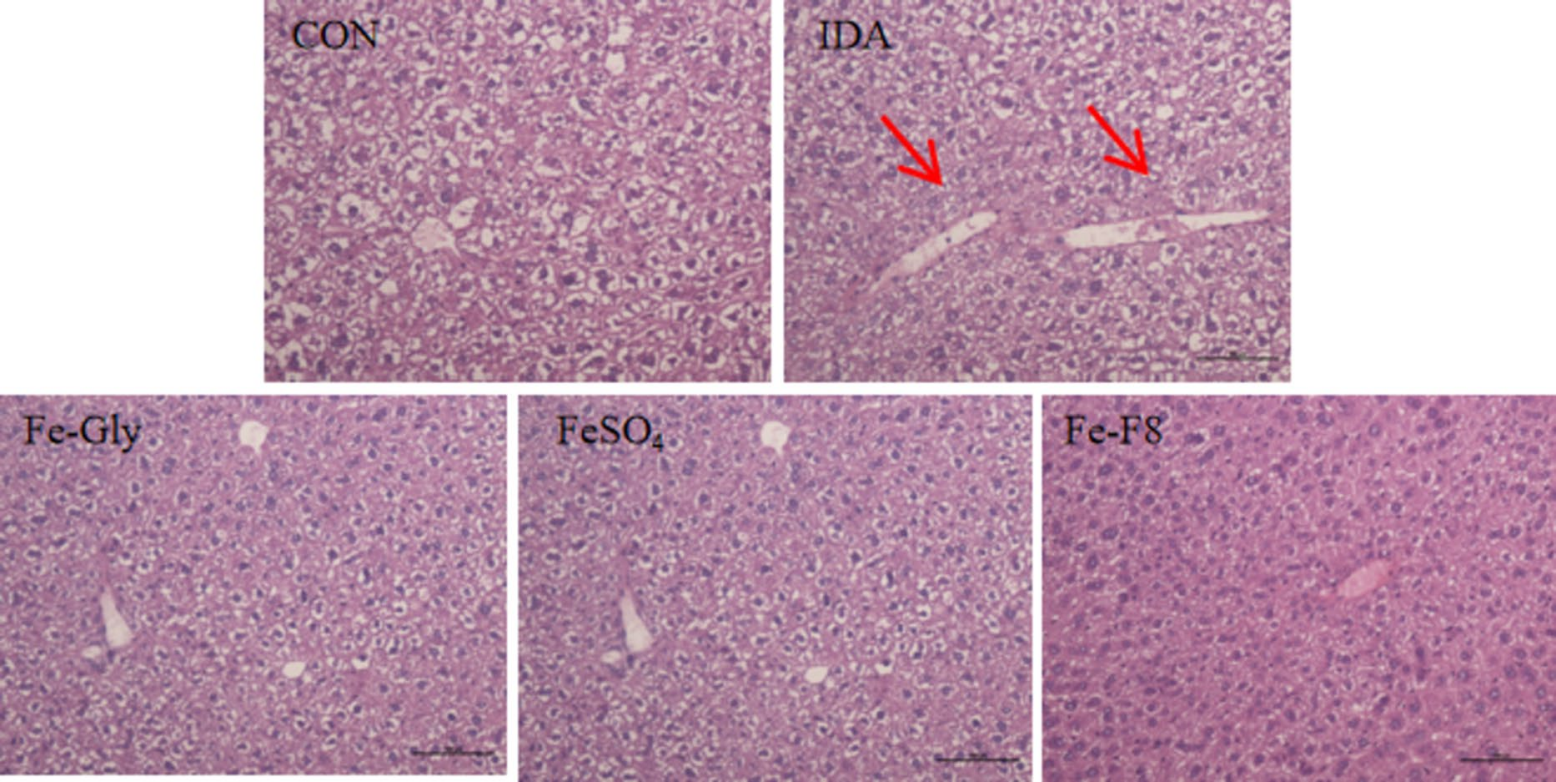
Liver morphology of mice in each group (H&E staining, 200× magnification)

Figure 3 the results of liver morphology of mice in each group showed that, compared with the normal control group, the hepatocyte cords around the central vein of the liver in IDA group were not clearly demarcated and arranged in a disorderly manner. After 4w of intragastric administration, the liver color of Fe-F8 group gradually turned red, and the lines of hepatocyte cords around the central vein were clear, and the arrangement tended to be neat. However, the cells in Fe-Gly group and FeSO_4_ group were improved to a certain extent, but the cord boundaries of hepatocytes were still unclear and the arrangement was chaotic. In conclusion, supplementation with a certain dose of Fe-F8 can significantly improve the damage of intestinal mucosal barrier and inflammatory cell infiltration caused by IDA, as well as the arrangement of liver cells, and the effect is better than Fe-Gly and FeSO_4_.

### Hb level, RBC counts, HCT and MCV levels

**Fig. 4:**
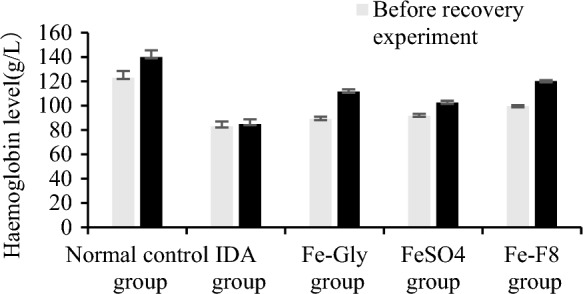
The hemoglobin changes of mice in different groups before and after the recovery experiment. The IDA group was not treated, normal control group was treated with standard granule diet, Fe-Gly group was treated with ferrous glycine (3 mg iron), FeSO_4_ group was treated with ferrous sulfate (3 mg iron), and Fe-F8 group was treated with yeast iron (3 mg iron), respectively

Hemoglobin levels before and after the trial are shown in Fig. 4. Hemoglobin is a red blood cell protein responsible for transporting oxygen into tissues, where iron has a central place in the hemoglobin structure, IDA can cause hemoglobin to decline (Linberg et al. 2009).

Due to iron deficiency diet, the hemoglobin content of the model group was the lowest, which was significantly lower than that of the normal control group (p < 0.05), Hb 83–84 g/L, belonging to the anemia level (less than 90 g/L).After feeding iron supplement (Fe-Gly, FeSO_4_ and Fe-F8) for 4w, the hemoglobin level of the three iron supplement groups recovered, and the Hb content of the three iron supplement groups had no significant difference compared with the normal control group at the beginning of the experiment (p > 0.05).Among them, the hemoglobin content of Fe-F8 group can be restored to the original level of normal control group, and the results confirmed that *S. cerevisiae* enriched iron can promote the recovery of hemoglobin content.

Hemoglobin is a pigment protein present in red blood cells. The main physiological function of RBC is realized through hemoglobin, transporting oxygen to the body’s tissue cells to produce energy for metabolism. Hematocrit (HCT) reflects the proportion of red blood cells in the whole blood (Chen et al. 2021).When iron intake is insufficient to meet daily needs, the body’s iron stores drop too low to support normal red blood cell production, resulting in IDA, followed by low HCT, MCV, MCH, or MCHC (Xiao et al. 2015).

The data in Table 5 show that after 4 weeks of recovery experiment, Hb of the anemia mice supplemented with iron-enriched *S. cerevisiae* recovers to 120.21 g/L, which is significantly different from that of the other two iron supplement groups, while the hemoglobin content of the Fe-Gly and FeSO_4_ groups is still lower than that of the normal control group. In addition, at the end of this experiment, RBC, HCT and MCV parameters of Fe-Gly group, FeSO_4_ group and Fe-F8 group were significantly higher than those of IDA group (p < 0.05). It is worth noting that RBC, HCT and MCV levels in Fe-F8 group were significantly higher than those in Fe-Gly group and FeSO_4_ group, indicating that Fe-F8 was more effective in improving anemia symptoms.

**Table 5:** Hematological parameters of mice in different groups

Group	Hb (g/L)	RBC (*10^12^*L^−1^)	HCT (%)	MCV (fL)
Normal control group	139.93 ± 5.56^a^	7.03 ± 0.61^bc^	40.24 ± 2.13^b^	70.12 ± 5.19^c^
IDA group	84.91 ± 3.89^e^	6.43 ± 0.47^c^	30.12 ± 1.94^e^	64.42 ± 4.38^d^
Fe-Gly group	111.57 ± 3.81^c^	7.91 ± 0.37^ab^	36.27 ± 2.21^c^	72.36 ± 6.01^b^
FeSO_4_ group	102.55 ± 4.13^d^	7.01 ± 0.24^c^	34.23 ± 2.72^d^	69.19 ± 4.51^c^
Fe-F8 group	120.21 ± 6.74^b^	8.04 ± 0.53^a^	43.88 ± 3.10^a^	75.27 ± 6.18^a^

*The superscript of the data in the table was obtained through ANOVA test analysis, and different letter superscripts indicated significant differences in the measured substance content (p < 0.05). The IDA group was not treated, normal control group was treated with standard granule diet, Fe-Gly group was treated with ferrous glycine (3 mg iron), FeSO_4_ group was treated with ferrous sulfate (3 mg iron), and Fe-F8 group was treated with yeast iron (3 mg iron), respectively

### Serum iron level

**Fig. 5:**
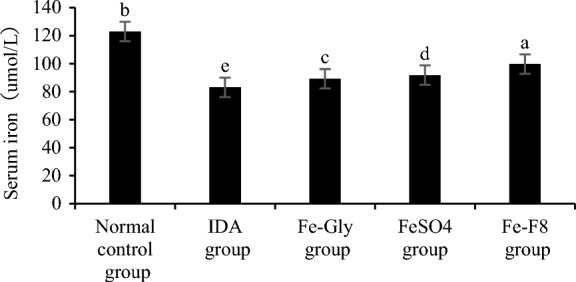
Serum iron levels in different groups. Data are presented as mean ± SD. Different letters show statistically significant differences (p < 0.05). The IDA group was not treated, normal control group was treated with standard granule diet, Fe-Gly group was treated with ferrous glycine (3 mg iron), FeSO_4_ group was treated with ferrous sulfate (3 mg iron), and Fe-F8 group was treated with yeast iron (3 mg iron), respectively

The changes of serum iron were shown in Fig. 5. The serum iron concentration in IDA group was the lowest and the highest in Fe-F8 group, which was significantly different from Fe-Gly group and FeSO_4_ group (p < 0.05). In this study, the SI level in the IDA group was significantly lower than that in the normal control group (p < 0.05), which was similar to the result observed by Xiao et al. (2015). The results showed that the *S. cerevisiae* enriched iron could promote the recovery of serum iron content.

### Immunoglobulin level

Immunoglobulins are major players in humoral immunity. IgG is the most abundant immunoglobulin in the body, which is mainly responsible for neutralizing toxins and regulating immune cell function, while IgM is mainly distributed in the blood and is the first antibody to appear in the initial immune response (Wang and Sun 2014). After 4 weeks of iron supplementation, the contents of immunoglobulin IgG and IgM in the blood of mice were measured by anatomic blood collection, and the results were shown in Table 6.

**Table 6:** The changes of serum immunoglobulin content in mice

Group	IgG (g/L)	IgM (g/L)
Normal control group	17.59 ± 0.48^c^	3.16 ± 0.12^d^
IDA group	11.76 ± 0.62^e^	2.65 ± 0.08^e^
Fe-Gly group	22.55 ± 2.12^d^	4.72 ± 0.19^c^
FeSO_4_ group	14.93 ± 0.31^b^	5.01 ± 0.23^b^
Fe-F8 group	24.68 ± 2.37^a^	6.06 ± 0.29^a^

*The superscript of the data in the table was obtained through ANOVA test analysis, and different letter superscripts indicated significant differences in the measured substance content (p < 0.05). The IDA group was not treated, normal control group was treated with standard granule diet, Fe-Gly group was treated with ferrous glycine (3 mg iron), FeSO_4_ group was treated with ferrous sulfate (3 mg iron), and Fe-F8 group was treated with yeast iron (3 mg iron), respectively

The IgG and IgM contents of mice in IDA group were significantly lower than those in Normal control group (p < 0.05). The IgG and IgM contents in iron supplementation group were significantly different from those in Normal control group, indicating that supplementation with a certain dose of iron could improve the immune status of IDA mice, and it was noteworthy that Fe-F8 group was the highest (p < 0.05).

### Antioxidant activity

#### Total antioxidant capacity

**Fig. 6:**
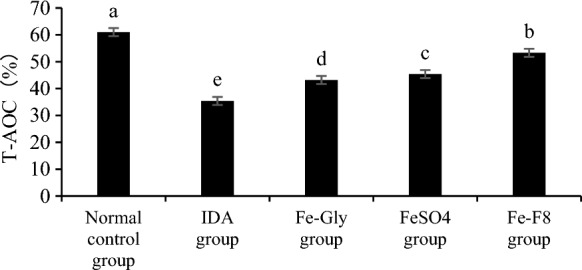
Effects of different treatments on total antioxidant capacity of mice. IDA group was not treated, normal control group was treated with standard granule diet, Fe-Gly group was treated with ferrous glycine (3 mg iron), FeSO_4_ group was treated with ferrous sulfate (3 mg iron), and Fe-F8 group was treated with yeast iron (3 mg iron), respectively

The total antioxidant activity and MDA levels of mice in each group were shown in Fig. 6 and Table 6. The results showed that compared with the model group, the *S. cerevisiae* enriched iron could improve the total antioxidant capacity in serum of mice. The total antioxidant capacity of yeast group was 53.31%, which was higher than that of other experimental groups, but slightly lower than that of Normal control group.

#### Antioxidant enzyme activity and MDA level

Table 7 compared with normal control group or iron supplement group, the activities of SOD and GSH-Px in IDA group were significantly decreased (p < 0.05), and the level of MDA in IDA group was also the lowest. In addition, the activities of SOD and GSH-Px in Fe-F8 group were higher than those in other iron supplement groups (p < 0.05), and the activities of SOD and GSH-Px in all groups were the highest. However, the MDA concentration of FeSO_4_ group was not significantly different from that of IDA group, and the MDA level of Fe-F8 group was higher than that of other groups. However, the results combined with SOD and GSH-Px levels showed that the influence of Fe-F8 group on serum antioxidant activity was more significant than that of Fe-Gly and FeSO_4_ group. Tang’s research results show that compared with FeSO_4_, heme iron-enriching peptides can enhance the activity of antioxidant (Tang et al. 2014).

**Table 7:** The activities of antioxidant enzymes and the MDA level of mice in different groups

Group	SOD (U/mL)	GSH-PX (U/mL)	MDA (umol/mL)
Normal control group	151.90 ± 3.53^d^	598.84 ± 24.49^c^	24.98 ± 2.45^b^
IDA group	100.55 ± 2.34^e^	464.77 ± 23.23^d^	16.07 ± 1.91^c^
Fe-Gly group	187.90 ± 4.25^b^	662.74 ± 28.13^b^	17.55 ± 1.77^c^
FeSO_4_ group	157.80 ± 3.57^c^	599.94 ± 29.08^c^	16.87 ± 1.19^c^
Fe-F8 group	198.06 ± 8.61^a^	669.90 ± 30.17^a^	35.51 ± 3.07^a^

*The superscript of the data in the table was obtained through ANOVA test analysis, and different letter superscripts indicated significant differences in the measured substance content (p < 0.05). The IDA group was not treated, normal control group was treated with standard granule diet, Fe-Gly group was treated with ferrous glycine (3 mg iron), FeSO_4_ group was treated with ferrous sulfate (3 mg iron), and Fe-F8 group was treated with yeast iron (3 mg iron), respectively

## Conclusions

In this study, a strain no. F8 with iron-rich potential was selected. On this basis, the improvement effect of Fe-F8 on iron-deficiency anemia was studied by using the iron deficiency anemia model of mice. Our results show that Fe-F8 can improve IDA in mice by weight, hematological parameters, organ parameters and antioxidant activity in vivo, we found that Fe-F8 has a more significant effect on IDA, with higher bioavailability and fewer side effects compared to Fe-Gly and FeSO_4_. This finding suggests that Fe-F8 has great potential as an effective source of iron supplementation in IDA mice, and the effects of strain no. F8 on other animals or humans need to be further studied. We studied the effect of iron deficiency on colon tissue, and we can continue to explore the specific mechanism of bacterial flora on intestinal inflammation in the later stage. The clinical significance of iron deficiency anemia is worthy of attention and further research. In addition, *S. cerevisiae* enriched iron still faces the problem of high preparation cost, and the optimization of fermentation process can be explored in the future to prepare iron supplements with similar activity but cheap.

## References

[CR1] Benoist B, McLean E, Egll I (2008). Worldwide prevalence of anaemia 1993–2005: WHO global database on anaemia.

[CR2] Bovellbenjamin AC, Viteri FE, Allen LH (2000). Iron absorption from ferrous bisglycinate and ferric trisglycinate in whole maize is regulated by iron status. Am J Clin Nutr.

[CR3] Cancelo-Hidalgo MJ, Castelo-Branco C, Palacios S (2013). Tolerability of different oral iron supplements: a systematic review. Curr Med Res Opin.

[CR4] Chaud MV, Izumi C, Nahaal Z (2002). Iron derivatives from casein hydrolysates as a potential source in the treatment of iron deficiency. J Agric Food Chem.

[CR5] Chen C, Zhou Y, Dong Y (2021). Diagnostic value of MCV, RDW, MCH and MCHC combined with trace elements iron, zinc and copper in children with iron deficiency anemia. Exp Lab Med.

[CR6] Dallman PR (2003). Biochemical basis for the manifestations of iron deficiency. Ann Rev Nutr.

[CR7] Dewey KG, Domello FM, Cohen RJ (2002). Iron supplementation affects growth and morbidity of breast-fed infants: results of a randomized trial in Sweden and Honduras. J Nutr.

[CR8] Duque X, Martinez H, Vilchis-Gil J (2014). Effect of supplementation with ferrous sulfate or iron bis-glycinate chelate on ferritin concentration in Mexican schoolchildren: a randomized controlled trial. Nutr J.

[CR9] Fu-Rong W, Zhong-Guo X (2014). Effectiveness of treatment of iron deficiency anemia in rats with squid ink melanin-Fe. Food Funct.

[CR10] Geng Q (2014). Culture of iron-rich yeast cells and its application in the treatment of iron-deficiency anemia.

[CR11] Georgieff MK (2011). long-term brain and behavioral consequences of early iron deficiencyn. Nutr Rev.

[CR12] Hassan TH, Badr MA, Karam NA (2016). Impact of iron deficiency anemia on the function of the immune system in children. Medicine.

[CR13] Hyder SM, Persson LA, Chowdhury AM (2002). Do side-effects reduce compliance to iron supplementation? A study of daily- and weekly-dose regimens in pregnancy. J Health Popul Nutr.

[CR14] Kyyaly MA, Powell C, Ramadan E (2015). Preparation of iron-enriched baker’s yeast and its efficiency in recovery of rats from dietary iron deficiency. J Nutr.

[CR15] Linberg R, Conover CD, Shum KL (2009). Hemoglobin based oxygen carriers: how much methemoglobin is too much?. Artif Cell Blood Substi Biotechnol.

[CR16] Lozoff B, Beard J, Connor J (2006). Long-lasting neural and behavioral effects of iron deficiency in infancy. Nutr Rev.

[CR17] Miquel E, Farré R (2007). Effects and future trends of casein phosphopeptides on zinc bioavailability. Trends Food Sci Technol.

[CR18] Paš M, Piškur B, Šuštarič M (2007). Iron enriched yeast biomass—a promising mineral feed supplement. Biores Technol.

[CR19] Souza AI, Filho MB, Bresani CC, Ferreira LOC, Figueiroa JN (2009). Adherence and side effects of three ferrous sulfate treatment regimens on anemic pregnant women in clinical trials. Cad Saude Publica.

[CR20] Tang N, Chen LQ, Zhuang H (2014). Effects of heme iron enriched peptide on iron deficiency anemia in rats. Food Funct.

[CR21] Wang M (2013). Effect of dietary oligopeptide iron on iron deficiency anemia in rats.

[CR22] Wang X, Sun L (2014). Effect of iron supplementation on immune function in patients with iron deficiency anemia. Shandong Med.

[CR23] Wang F, Zhao W, Chen J (2016). Research progress of iron supplement. Adv Pharm.

[CR24] Wang M, Wan P, Zhu L (2019). Colon injury induced by iron deficiency anemia (IDA) in mice. Natl Sci J Heilongjiang Univ China.

[CR25] Xiao C, Lei X, Wang Q (2015). Effects of a tripeptide iron on iron-deficiency anemia in rats. Biol Trace Elem Res.

[CR26] Xue D, Zhang H, Zhao X (2003). Fermentation culture and nutritional evaluation of ferric nutritive yeast. Food Sci.

[CR27] Yuan Y, Guo X, He X (2004). Construction of a high-biomass, iron-enriched yeast strain and study on distribution of iron in the cells of *Saccharomyces cerevisiae*. Biotech Lett.

[CR28] Yun S, Zhang T, Li M (2011). Proanthocyanidins inhibit iron absorption from soybean (*Glycine max*) seed ferritin in rats with iron deficiency anemia. Plant Foods Hum Nutr.

[CR29] Zhang X, Hu Z, Luo Y (2004). Pharmacokinetics and bioavailability of iron-rich yeast. Chin J Veter Med China.

[CR30] Zhang XG, Peng YN, Li XR (2015). Screening of iron-enriched fungus from natural environment and evaluation of organically bound iron bioavailability in rats. Food Sci Technol.

[CR31] Zhang XG, Wei GX, Wang WN, Ma GD, Tang P, Chen XQ (2016). Effects of Fe-YM1504 on iron deficiency anemia in rats. Food Funct.

[CR32] Zhang XG, Wang N, Ma GD (2021). Preparation of S-iron-enriched yeast using siderophores and its effect on iron deficiency anemia in rats. Food Chem.

[CR33] Zheng Y, Li X, Xie Y (2001). Effect of iron deficiency anemia on the reproduction of female rats and the development of their offspring. J Hyg Res.

